# Biocompatible Nano-Hydroxyapatites Regulate Macrophage Polarization

**DOI:** 10.3390/ma15196986

**Published:** 2022-10-08

**Authors:** Da-Wang Zhao, Xin-Cheng Fan, Yi-Xiang Zhao, Wei Zhao, Yuan-Qiang Zhang, Ren-Hua Zhang, Lei Cheng

**Affiliations:** 1 Department of Orthopedics, Qilu Hospital of Shandong University, Jinan 250012, China; 2 Institute of Stomatology, Shandong University, Jinan 250012, China; 3 Department of Orthopaedics, Taian City Central Hospital, Tai’an 271000, China; 4 Outpatient Department, Qilu Hospital of Shandong University, Jinan 250012, China

**Keywords:** hydroxyapatites, immunomodulation, inflammatory response, macrophage

## Abstract

Research on regulation of the immune microenvironment based on bioactive materials is important to osteogenic regeneration. Hydroxyapatite (HAP) is believed to be a promising scaffold material for dental and orthopedic implantation due to its ideal biocompatibility and high osteoconductivity. However, any severe inflammation response can lead to loosening and fall of implantation, which cause implant failures in the clinic. Morphology modification has been widely studied to regulate the host immune environment and to further promote bone regeneration. Here, we report the preparation of nHAPs, which have uniform rod-like shape and different size (200 nm and 400 nm in length). The morphology, biocompatibility, and anti-inflammatory properties were evaluated. The results showed that the 400 nm nHAPs exhibited excellent biocompatibility and osteoimmunomodulation, which can not only induce M2-phenotype macrophages (M2) polarization to decrease the production of inflammatory cytokines, but also promote the production of osteogenic factor. The reported 400 nm nHAPs are promising for osteoimmunomodulation in bone regeneration, which is beneficial for clinical application of bone defects.

## 1. Introduction

The implantation of bone biomaterials has been recognized as a promising method for the treatment of bone defects clinically. However, prosthesis loosening still accounts for more than half of implant failures [1,2]. This phenomenon is mainly attributed to insufficient osseointegration caused by inflammatory response. Tissue engineering has placed significant focus on designing biomaterial scaffolds capable of directing the fate of target cells. Several studies have demonstrated that the specific concentrations and specific morphology of designed biomaterials can affect mesenchymal stem cell (MSC) differentiation and promote successful tissue regeneration in orthopedics and dentistry [3,4,5,6]. In addition to component construction, they are also working to influence the interaction between stem cells and immune cells (macrophages) in the body through biomaterial designing, because this interaction between cells will affect the regeneration and repair ability of progenitor cells [7]. Although it is necessary to consider the immunological effects of biomaterials in the body [8], there is still very little work that can focus on the design of biomaterials that reduce the immune response in vivo and promote tissue regeneration and repair.

When the biomaterial is implanted in the body, it will cause the aggregation of macrophages and induce an immune response [9]. Furthermore, macrophages will be polarized into two forms, including M1-phenotype macrophages (M1) and M2-phenotype macrophages (M2). In the bone regeneration stage, the appropriate dose of inflammatory factors (M1 related cytokines TNFα) can promote fracture healing [10]. However, the long-term sustained M1 environment will produce bone destruction and is not conducive to bone regeneration and repair [11]. In this case, alternatively activated M2 polarization can promote bone formation and tissue repair. Nanoparticle-mediated M2 macrophage polarization enhances bone formation and osteogenesis [12]. Strontium-zinc phosphate chemical conversion coating improves the osseointegration of titanium implants by regulating M2 polarization [5]. The hierarchical nanointerface possesses the capacity to recruit host MSCs and promote endogenous bone regeneration by immunomodulation of macrophage polarization [13]. Overall, the above studies have shown that when designing biomaterials, considering the issue of osteoimmunomodulation will provide better osteogenesis and tissue repair effects.

Osteoimmunomodulation can be modulated through material design in several ways. For example, the morphology and composition of the biomaterials can affect the osteogenic differentiation of MSCs [5]. Additionally, the micro-nanostructure of hydroxyapatite [HAP, Ca_10_(PO_4_)_6_(OH)_2_] has been proven to be the main inorganic constituent of animal bones with exceptional biocompatibility, appropriate biodegradability, and high osteoinductivity. This indicates the excellent structural and functional similarity to the mineral composition of natural bones [14,15,16]. Several properties of HAP maybe affect the cell function, such as shape, X-ray radiation, temperature, surface charges and so on [17]. Some researchers find that Nano-HAPs are defined as 100–500 nm long and 30–50 nm wide rod-like structures, which have better effects than granular structures in “stirring” organelles to stimulate cell function. Furthermore, they are more easily internalized by cells than linear structures. However, the effect of micro-nano HAP on macrophage polarization and immune microenvironment has not been studied. Here, we demonstrated that 400 nm nHAPs can promote the polarization of M2 and verified the related gene changes and signaling pathways through KEGG pathways and GO terms. New methods have been used in biomaterial modification to improve the nature of biomaterials and affect the immune microenvironment to achieve tissue repair capabilities [18,19]. In this work, we investigated the mechanism of nHAP-induced M2 regulation and found that 400 nm nHAPs can promote M2 polarization.

## 2. Materials and Methods

### 2.1. Materials

Oleic acid, CaCl_2_, and NaH_2_PO_4_·2H_2_O were obtained from Sinopharm (China National Pharmaceutical Group Corporation, Shanghai, China). APC anti-rat CD11b/c (BioLegend Global Headquarters, San Diego, CA, USA), M-CSF (PeproTech, Cranbury, NJ, USA).

### 2.2. Methods

#### 2.2.1. Fabrication and Morphology of nHAP

The 200 nm and 400 nm nHAPs were fabricated according to a previous report [16]. Briefly, the instructions are as follows: mix 8 mL of oleic acid and 16 mL (20 mL for 200 nm nHAP) of ethanol, then add 6 mL (4 mL for 200 nm nHAP) of NaOH solution, 176 mg CaCl_2_ solution, 230 mg NaH_2_PO_4_·2H_2_O solution, respectively, place under vigorous stirring, and stir for 15 min. Subsequently, the reaction system was moved into a 180 °C reactor and for 12 h. The products were washed six times in ethanol and double distilled water, respectively. The morphologies of the 200 nm and 400 nm nHAPs were observed by scanning electron microscopy (SEM, Zeiss G300, Oberkochen, Germany). The nHAPs were then dispersed in PBS solution with the dilution of 100 µg/mL for further biological experiments.

#### 2.2.2. Rat Macrophage Isolation, Culture, and Identification

The isolation, culture, and characterization of rat macrophages were performed using a previously published method [5]. Briefly, cells obtained from the femur of Sprague-Dawley male rats were cultured with 30 ng/mL of M-CSF in DMEM (Gibco, Waltham, MA, USA) medium, and the cells cultured after 1 week were regarded as primary macrophages. We detected the cell surface marker CD11b by flow cytometry, and CD11b-positive cells were regarded as macrophages.

#### 2.2.3. Cell Apoptosis

To evaluate the biocompatibility of nHAPs, cell apoptosis and necrosis were analyzed using Gallios flow cytometer (Beckman, Brea, CA, USA), according to manufacturer’s protocols with Annexin V and 7-AAD staining on the first day [20].

#### 2.2.4. Methyl Thiazolyl Tetrazolium (MTT) Assay

After macrophages were co-cultured with nHAP for 0, 2, 4, 6, and 8 days, the cell proliferation was measured by MTT Cell Proliferation Assay Kit (Solarbio, Beijing, China). At each time point, the original medium was extracted, and 100 μL of MTT solution and 900 μL DMEM were added. Finally, 500 μL of formazan was added and the absorbance was measured at 490 nm in triplicate to quantify cell viability.

#### 2.2.5. Live/Dead Staining

The cells were washed twice with Assay Buffer and subsequently stained with Calcein AM/PI working solution and observed under a confocal microscope (Opera Phenix, PerkinElmer, Waltham, MA, USA).

#### 2.2.6. Reverse Transcription-Polymerase Chain Reaction (RT-PCR)

After culturing in with nHAPs in the six-well plate for two days, quantitative analysis of the target mRNA expression was performed with RT-PCR. Total RNA was reverse-transcribed and amplified in triplicate using reverse transcriptase ReverTra Ace (Toyobo, Osaka, Japan) with a real-time PCR machine (Applied Biosystems, Waltham, MA, USA), according to the manufacturer’s instructions. β-actin was regarded as a housekeeping gene. The following are the primer sequences:

CD206: Forward 5′-GAGGACTGCGTGGTGATGAA-3′ and reverse 5′-CATGCCGTTTCCAGCCTTTC-3′;
Arg1: Forward 5′-AAGACAGGGCTACTTTCAGGAC-3′ and reverse 5′-ACCTTCCCGTTTCGTTCCAA-3′;
IL10: Forward 5′-TAACTGCACCCACTTCCCAG-3′ and reverse 5′-TGGCAACCCAAGTAACCCTTAAA-3′;
β-actin: Forward 5′-CCTCTATGACAACACAGT-3′ and reverse 5′-AGCCACCAATCCACACAG-3′.

#### 2.2.7. RNA-Sequence

To evaluate the effect of 400 nm nHAP on the genes of macrophages, we used RNA-sequence to evaluate gene changes, signal pathway analysis and so on. Macrophages were cultured with 400 nm nHAP, and after 3 days, total mRNA was collected for RNA-sequence analysis by the Lian Chuan Biotechnology Institute (LC, Hangzhou, China). We used: volcano diagrams to analyze the overall impact of 400 nm nHAP on macrophage genes; Kyoto Encyclopedia of Genes and Genomes (KEGG) pathways to analyze the change index of macrophage polarization-related genes; the GO terms to analyze the enrichment of genes related to biological functions, and further analyze up and down regulation of signaling pathways.

#### 2.2.8. Statistical Analysis

Data are shown as the mean ± standard deviation from at least three independent experiments. Differences among groups were analyzed with one-way ANOVA by SPSS Statistics 23. * and # represent *p* < 0.05 compared with control and 200 nm nHAP, respectively; ** and ## represent *p* < 0.01 compared with control and 200 nm nHAP, respectively.

## 3. Results

### 3.1. Characterization and Biocompatibility of nHAPs

**Figure 1:**
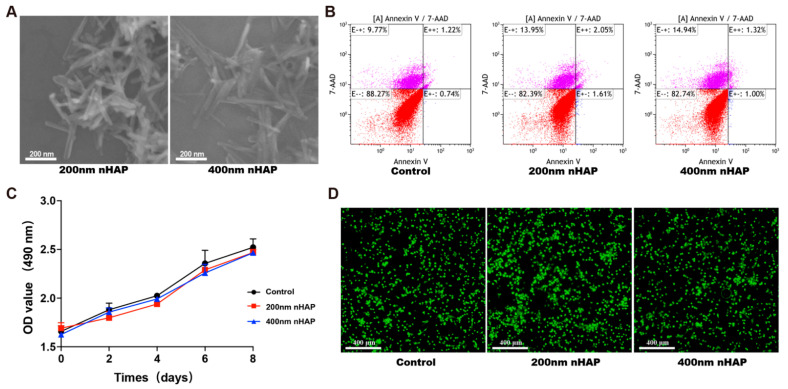
Characterization and biocompatibility of the nHAPs. (**A**) The SEM images of nHAP. (**B**) Percentages of apoptotic macrophages cultured with nHAP using Annexin V-7-AAD staining determined by flow cytometry. (**C**) Cell viability of macrophages cultured for 2, 4, 6, and 8 days with nHAP by MTT assay. (**D**) Live and Dead staining of Macrophages.

The morphology of the nHAPs constructed by a hydrothermal method was studied. The scanning electron microscope image shows that the 400 nm nHAPs (≈400–450 nm in length, ≈30–40 nm in width) and 200 nm nHAPs (≈150–200 nm in length, ≈30–40 nm in width) were uniform (Figure 1A). Biocompatibility is a prerequisite for material application. To evaluate the biocompatibility of nHAPs, we cultured macrophages in DMEM medium containing 100 µg/mL nHAPs in 6-well plate for 1 day, and then performed apoptosis test through flow cytometry. The ratios of cells in the early stages of apoptosis are 0.74% (control), 1.61% (200 nm nHAP), and 1.00% (400 nm nHAP). In addition, the ratios of necrotic cells are 1.22% (control), 2.05% (200 nm nHAP), and 1.32% (400 nm nHAP) (Figure 1B). Cell proliferation was calculated on the specific timepoint (0, 2, 4, 6, and 8 days) by MTT assay. The results in Figure 1C showed there was no difference in the proliferation rate between different sizes of nHAP. From the images of Live/Dead staining, no dead cells were seen in the 200 nm or 400 nm nHAP group, which showed good biocompatibility (Figure 1D). From the results, a good biocompatibility was shown in 200 nm and 400 nm nHAP groups in vitro.

### 3.2. Evaluation of Macrophage Characterization and Polarization

**Figure 2:**
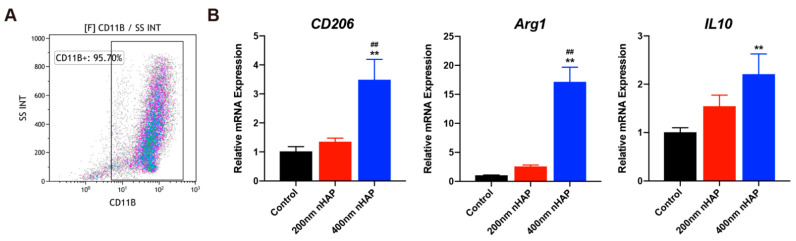
400 nm nHAP regulates macrophage polarization. (**A**) Percentages of CD11b-positive macrophages by flow cytometry. (**B**) RT-PCR results for CD206, Arg1 and IL10 mRNA in macrophages on day 2 with nHAP. (** and ## represent *p* < 0.01 compared with control and 200 nm nHAP, respectively).

We first identified macrophages by flow cytometry, and CD11b positive cells were considered macrophages. High-purity (95.70%) macrophages ensure the subsequent experiments (Figure 2A). Macrophages undergo two different polarization states: the classically activated M1 and the alternatively activated M2 [21]. In general, M1 have been found to exacerbate tissue injury. However, M2 take part in polarized Th2 responses, parasite clearance, anti-inflammation, tissue remodeling, angiogenesis, and immunoregulation [22,23,24].

Macrophages (5 × 10^4^ /mL) were cultured in a 24-well plate with nHAPs for 2 days. RT-PCR was performed to detect the expression of M1 and M2-related markers. As shown in Figure 2B, expression of the M2 surface marker *CD206* was significantly increased in 400 nm nHAP group at 2 days. Meanwhile, there was a clear enhancement of the M2-related cytokine (Arg1 and IL10) in 400 nm nHAP group, which indicated that 400 nm nHAP can promote M2 polarization. This has been shown to be beneficial for tissue repair and osteogenic regeneration [12].

### 3.3. RNA-Sequence

**Figure 3:**
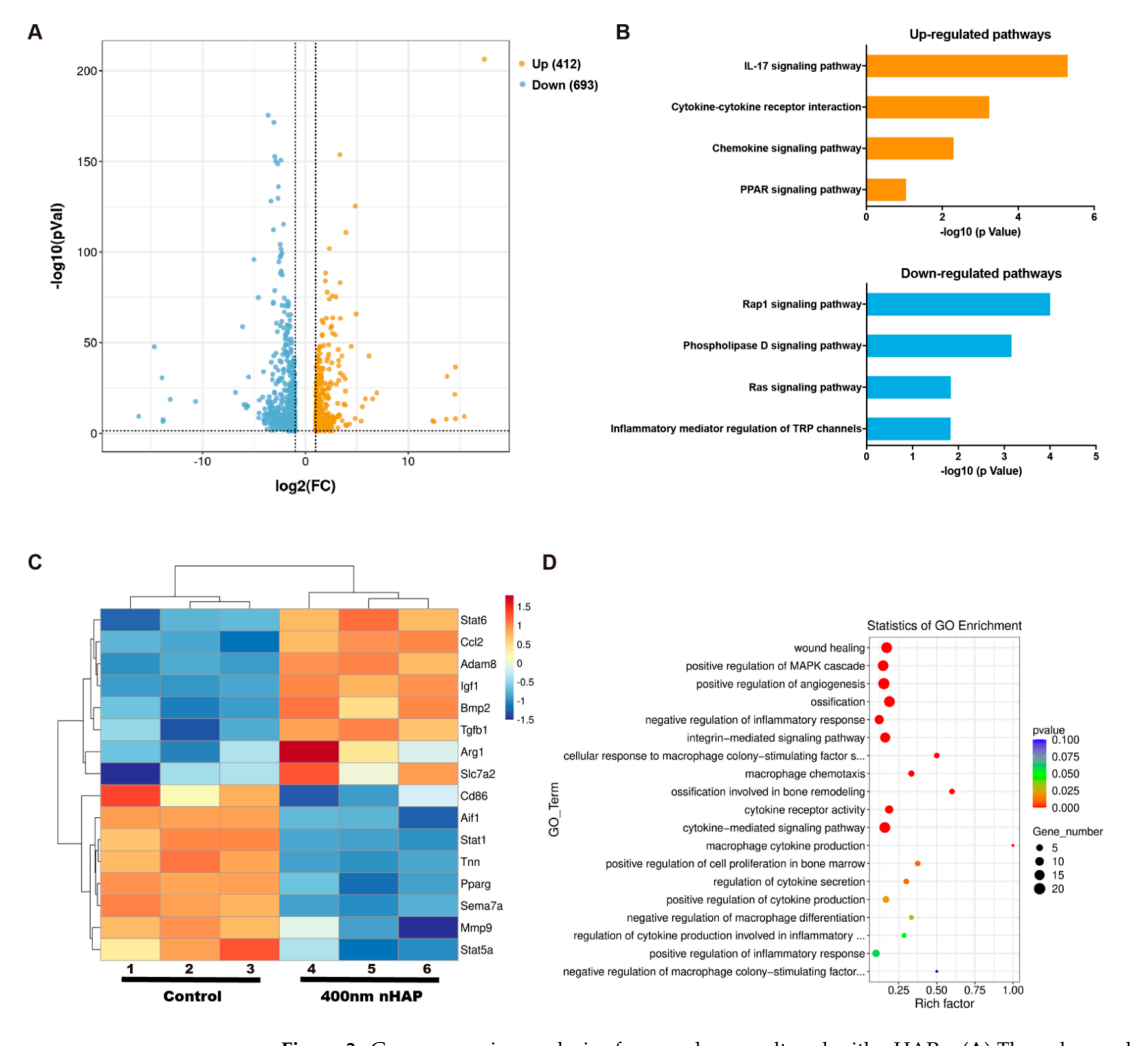
Gene expression analysis of macrophages cultured with nHAPs. (**A**) The volcano plot of differential expression of genes. (**B**) Representative top 4 upregulated and 4 downregulated pathways analyzed by KEGG pathway method. (**C**) Microarray heat map depicting the fold change in expression of selected genes. (**D**) Statistics of GO Enrichment.

To explore the mechanism related to the functions of M2-like activation by 400 nm nHAPs, we performed transcriptome analysis by high-throughput RNA sequencing (RNA-Seq) using triplicates of macrophages after culturing with 400 nm nHAPs. As shown in Figure 3A, 412 genes were up-regulated while 693 genes were downregulated in the 400 nm nHAP group compared to control group. KEGG pathway analysis showed that the IL-17 signaling pathway was markedly up-regulated in 400 nm nHAPs cultured macrophages compared to the control group, followed by Cytokine–cytokine receptor interaction, Chemokine, and PPAR signaling pathways. Conversely, down-regulated signaling pathways including Rap1, Phospholipase D, Ras, and Inflammatory mediator regulation of TRP channels signaling pathways (Figure 3B).

Stat6 is known to drive macrophage M2 polarization [25]. The CCL2-CCR2 axis regulates macrophage polarization by influencing the expression of polarization-related genes and downmodulating proinflammatory cytokine production [26]. Classically activated M1 promotes the production of pro-inflammatory cytokines such as TNF-α, CCR7, iNOS, IL-1β, and IL-6, while M2 promotes the production of anti-inflammatory cytokines such as Arg1 and BMP2 [27]. In addition, Allograft inflammatory factor1 (AIF1) has been characterized as a pro-inflammatory molecule expressed in the macrophage [28]. From the up-regulated and down-regulated genes in volcano plot, we screened the genes related to M1/M2 polarization and made a heat map (Figure 3C). From the results of heat map, the Stat6, CCL2, ADAM8, IGF1, BMP2, TGF-β, Arg1, Slc7a2 were up-regulated in the 400-nm nHAP group. However, Stat1, Tnn, PPARγ, Sema7a, Stat5a, MMP9, IL-1β, Aif1, CD86, IL-6 were down-regulated in the 400 nm nHAP group compared to the control group. These results indicated that the 400 nm nHAPs induce the production of M2-related genes. Furthermore, the results of GO enrichment demonstrated that the 400 nm nHAPs are linked to functional networks involving the inflammatory response, cytokine production, ossification, etc. (Figure 3D). This finding supports the proposed importance of 400 nm nHAPs in osteoimmunomodulation.

## 4. Discussion

Studies have shown that immune cells interact with the skeletal system and play an important role in the repairing phase after tissue injury [29,30,31]. The inflammatory response reaches its peak within 24 h and releases pro-inflammatory cytokines, which can promote early blood vessel formation. Then it enters a recovery period, at which time intramembranous osteogenesis is formed [31,32]. During the recovery period, pro-inflammatory cytokines will be missing, and factors such as BMP2 play a role in promoting tissue repair [31,33]. An imbalance in immune regulation in the early fracture repairing stage, which is believed to break the repairing cascade and lead to undesirable bone repair. Therefore, adding factors that can regulate the immune response is regarded as a potential strategy to promote bone tissue repair.

In this study, we prepared different sizes of nHAP (200 nm and 400 nm in length), and verified its uniform and nanorod-like morphology in the material science characterization. From the results of the MTT assay and Live/Dead staining, we identified the excellent biocompatibility of the nHAPs in vitro, which indicated that nHAP had been successfully prepared. Its excellent biocompatibility provides a basis for later experiments. More recent research indicates that M2 polarization play an important role in bone mineralization and osteogenesis as well [34,35]. This result can be explained as, in part, by production of BMP2, transforming growth factor beta (TGFβ), and insulin like growth factor1 (IGF1) from M2 that promote osteogenesis differentiation [36]. From the results of Figure 2, we clarified that 400 nm nHAPs can induce M2 polarization and produce osteoinductive factor BMP2, which identifies that M2 can produce osteogenic cytokines and factors to create an immune microenvironment conducive to osteogenesis and tissue repair. We believe that some nHAPs induce macrophage polarization by releasing Ca^2+^, and others are phagocytosed into macrophages and play a regulatory role in cells.

Regarding the signaling pathways that can induce osteogenic differentiation, the cytokine-cytokine receptor interaction signaling pathways is also up-regulated in the 400 nm nHAPs group. However, in pro-inflammatory macrophages, Rap1 favors a pro-inflammatory environment and promotes cytokine production [37]. In addition, Phospholipase D is thought to participate in the autophagy function of macrophages [38]. Meanwhile, RAS and TRP channels were accompanied with inflammation response, and pro-inflammatory cytokines (IL-1β, IL-6, COX2 and PGE2) [39,40]. In this way, Rap1, Phospholipase D, Ras, and Inflammatory mediator regulation of TRP channels signaling pathways were down-regulated (Figure 3B) in 400 nm nHAPs, which indicated that 400 nm nHAPs can promote M2 polarization and down-regulate the related inflammatory response. The resulting M2 polarization produces more anti-inflammatory mediators, thereby promoting tissue repair. Furthermore, Arg1 and BMP2, which have been identified to induce osteogenesis of mesenchymal stem cells, are regarded as M2-related surface marker and osteogenic cytokine, respectively. [41,42]. The up-regulation of Arg1, BMP2, etc. (Figure 3C) indicates that 400 nm nHAPs can induce M2 polarization and anti-inflammatory cytokines, down-regulate the expression of IL-1β, and IL-6, thereby regulating the osteoimmuno-microenvironment and bone regeneration.

## 5. Conclusions

We first investigated the osteoimmunomodulatory property of 400 nm nHAPs. The in vitro experiments confirmed that 400 nm nHAPs can induce M2 polarization (an anti-inflammatory phenotype) with the production of anti-inflammatory and osteogenic cytokines. Our results indicate that the up-regulation of the IL-17 signaling pathway and the down-regulation of Rap 1 signaling pathway may be the most probable mechanisms underlying the immunomodulatory effects. This suggests that 400 nm nHAP is a promising and effective biomaterial for developing advanced bone regeneration and immunomodulation.

## Data Availability

The data collected for the study are available from the corresponding authors upon request.
